# Status and advances in mining for blackleg (*Leptosphaeria maculans*) quantitative resistance (QR) in oilseed rape (*Brassica napus*)

**DOI:** 10.1007/s00122-021-03877-0

**Published:** 2021-06-09

**Authors:** Junrey Amas, Robyn Anderson, David Edwards, Wallace Cowling, Jacqueline Batley

**Affiliations:** 1 School of Biological Sciences and The UWA Institute of Agriculture, The University of Western Australia, Perth, WA 6001 Australia; 2 School of Agriculture and Environment and The UWA Institute of Agriculture, The University of Western Australia, Perth, WA 6009 Australia

## Abstract

**Key message:**

Quantitative resistance (QR) loci discovered through genetic and genomic analyses are abundant in the *Brassica napus* genome, providing an opportunity for their utilization in enhancing blackleg resistance.

**Abstract:**

Quantitative resistance (QR) has long been utilized to manage blackleg in *Brassica napus* (canola, oilseed rape), even before major resistance genes (R-genes) were extensively explored in breeding programmes. In contrast to R-gene-mediated qualitative resistance, QR reduces blackleg symptoms rather than completely eliminating the disease. As a polygenic trait, QR is controlled by numerous genes with modest effects, which exerts less pressure on the pathogen to evolve; hence, its effectiveness is more durable compared to R-gene-mediated resistance. Furthermore, combining QR with major R-genes has been shown to enhance resistance against diseases in important crops, including oilseed rape. For these reasons, there has been a renewed interest among breeders in utilizing QR in crop improvement. However, the mechanisms governing QR are largely unknown, limiting its deployment. Advances in genomics are facilitating the dissection of the genetic and molecular underpinnings of QR, resulting in the discovery of several loci and genes that can be potentially deployed to enhance blackleg resistance. Here, we summarize the efforts undertaken to identify blackleg QR loci in oilseed rape using linkage and association analysis. We update the knowledge on the possible mechanisms governing QR and the advances in searching for the underlying genes. Lastly, we lay out strategies to accelerate the genetic improvement of blackleg QR in oilseed rape using improved phenotyping approaches and genomic prediction tools.

## Introduction

*Brassica* *napus* (oilseed rape/canola, AACC; 2n = 38) is a major source of oil for the human diet and industrial applications. After soybean, it is the second most important oilseed crop, with an estimated global production of over 76 million tons in 2017 (FAOSTAT 2018). Also, the oil-free meal is high in protein which is valuable for animal feed and potentially as a plant protein source of human food (Campbell et al. 2016). This amphidiploid was formed from the natural hybridization between the diploid species, *Brassica rapa* (AA) and *Brassica oleracea* (CC), occurring around ~ 7500 years ago (Chalhoub et al. 2014). Oilseed rape is considered an important commodity in many agricultural economies, including Canada, China, India and Australia, primarily cultivated under a monoculture system (FAOSTAT 2018). However, this continuous monoculture has encouraged the build-up of pathogen races capable of causing significant yield losses annually. Blackleg, caused by *Leptosphaeria maculans,* remains one of the most damaging diseases affecting global oilseed rape production. The disease has been documented to cause more than 10% in annual average yield loss with a maximum loss varying between 30 and 50%, depending on climatic conditions among oilseed rape growing countries (Hwang et al. 2016; Van de Wouw et al. 2016). The substantial economic damage brought by these losses makes blackleg a major threat to the global oilseed rape industry; hence, the development of control measures for this disease is a priority undertaking.

The causal organism, *L. maculans*, is a hemibiotrophic fungal pathogen capable of infecting the oilseed rape plant at all growth stages. The related species *L. biglobosa* is also becoming an important pathogen in some countries such as China and Canada (Cai et al. 2014; Fernando et al. 2016; Zhou et al. 2019a, b, c). The pathogen’s high evolutionary potential is due to its ability to produce both sexual (ascospores) and asexual (pycnidiospores) spores. Germ tubes from these spores can penetrate stomates or wounds, eventually colonizing the stem leading to crown canker (Rouxel et al. 2003a, b). Ascospores cause primary infections early in the growing season, and pycnidiospores cause multiple cycles of infection within the growing season (West et al. 2001). This alternate sexual and asexual propagation continues indefinitely because the fungus can survive dry or cold periods as a saprophyte in dead host tissue (Huang et al. 2007; Naseri et al. 2008). The fungus revives when environmental conditions are favourable, and produces the sexual fruiting body from which ascospores are released and infect suitable hosts. The ascospores are also windblown which aids long-distance transmission and can be continuously produced for several months, resulting in sufficient inoculum to infect plants in subsequent crops (Rouxel and Balesdent 2005; West et al. 2001).

Several approaches have been employed to manage blackleg. These include growing resistant cultivars, crop rotation, stubble management and judicious use of fungicides (West et al. 2001). Among these approaches, the deployment of genetic resistance has been considered the most sustainable approach and the cornerstone of blackleg management. In oilseed rape, two types of genetic resistance against blackleg have been widely recognized: qualitative or major (R) gene resistance and quantitative resistance (QR). Major gene resistance involves a molecular recognition between the resistance (R-gene) in the plant and the pathogen’s avirulence genes (*Avr*). This interaction prompts a cascade of molecular events to effect immunity (Larkan et al. 2013). Major gene resistance is initially manifested at the cotyledon stage and may extend towards the later stage (Raman et al. 2012a, b,^ 2016^). On the other hand, QR is controlled by several genes of modest genetic effects (Poland et al. 2009; Roux et al. 2014), providing partial and race non-specific protection (Brun et al. 2010). QR is mostly expressed at the adult plant stage and, hence, has also been called adult plant resistance (APR). R-gene-mediated resistance affords complete immunity to plants, whereas QR results in a reduction, rather than absence, of the disease (St.Clair 2010).

At least 18 major loci (R-genes) in *Brassica* have been identified that confer a gene-for-gene interaction with *L. maculans* (Delourme et al. 2004, 2006; Li and Cowling 2003; Long et al. 2011; Yu et al. 2005, 2008). Three of these genes (*Rlm2, LepR3* and *Rlm9*) have already been functionally tested and cloned (Larkan et al. 2013, 2015, 2020). Major R-genes are widely deployed in modern oilseed rape varieties and have been regarded as key components in the long-term viability of the oilseed rape industry (Salisbury et al. 2016). However, the continuous deployment of these genes has encouraged an evolutionary arms-race between pathogens and host plants; the large phenotypic effect of R-genes imposes strong selection pressure on the pathogen, causing it to evolve higher virulence through various mechanisms. Molecular investigations revealed mechanisms contributing to the pathogen’s evolution include mutation, deletion, inactivation, or down-regulation of the *Avr* gene leading to avoidance of specific *R-Avr* gene recognition and eventual breakdown of R-genes (Jones and Dangl 2006; Van de Wouw and Howlett 2020). These resistance breakdown events are characterized as a typical boom and bust cycle and have been documented in several instances including the outbreak following the breakdown of “sylvestris” resistance in Australia in 2003 (Sprague et al. 2006a, 2006b). Similar resistance breakdown events have also been recorded in France (Rouxel et al. 2003b) and Canada (Zhang et al. 2016). These epidemics provided evidence of R-genes’ vulnerability to breakdown and engendered exploration of the alternative QR which has been known to be more durable than qualitative resistance (Parlevliet 2002). Due to this, there is an increasing interest in QR’s utility for improving resistance towards blackleg.

In oilseed rape, QR for blackleg has traditionally been incorporated in cultivars through field selection and conventional breeding (Raman et al. 2020b). Field selection mainly relied on visual inspection of the phenotype, which is usually based on the overall crop stand or through estimation of stem canker infection and is evaluated at the end of the growing season (Huang et al. 2014). In Australia, canola breeding from 1970 to 2000 resulted in significant improvement in QR in commercial varieties over 30 years, despite evidence of erosion of QR after their release (Cowling 2007). However, unlike R-gene-mediated resistance, much of the underlying mechanisms controlling QR remain to be uncovered. Understanding the genetic control of blackleg QR has been mostly conducted by mapping quantitative trait locus (QTL) regions and associated molecular markers. These QTL mapping experiments are usually derived from bi-parental populations, mostly subjected to microspore culture to produce doubled-haploid populations (DH) (e.g. Raman et al. 2012a, b). Recently, the availability of high-quality molecular markers from the rapid development in next-generation sequencing has enabled the analysis of marker–trait association through genome-wide association studies (GWAS) in diversity panels (Fikere et al. 2018, 2020a, b; Fopa Fomeju et al. 2014; Jestin et al. 2011; Kumar et al. 2018; Raman et al. 2020a). These QTL mapping and association analyses revealed the abundance of genomic regions harbouring QR loci distributed throughout the *B. napus* genome (Raman et al. 2020a). However, most QR genomic regions display highly variable genetic effects and are highly influenced by environmental conditions (genotype by environment, GxE), making the study of QR challenging.

A range of *Brassica* germplasm has been explored as sources of QR. The original source of QR in Australian canola was moderately resistant Asian germplasm, introduced around 1970, which contributed 50.3% of the pedigrees of varieties released in 2000 (Cowling 2007). In Europe, the cultivar Darmor is the primary source of QR and its resistance alleles were proposed to have originated from one of its parents, Jet Neuf, which was extensively cultivated in Europe in the 1970s and the 1980s due to its high level of field blackleg resistance. These resistance alleles appeared to exhibit stability, as evidenced by their repeated detection in several mapping experiments across oilseed rape growing countries (Huang et al. 2016; Jestin et al. 2015, 2012; Kumar et al. 2018; Pilet et al. 2001; Raman et al. 2018). With the increased interest in QR, several other cultivars have also been explored in several QTL mapping and GWAS experiments, resulting in the discovery of several loci underlying blackleg QR (Li and Cowling 2003; Yu et al. 2005, 2008; Kaur et al. 2009; Long et al. 2011; Raman et al. 2012b, 2020b; Larkan et al. 2016).

Advances in genomics have provided the tools to further analyse QR for its utilization in crop breeding. The availability of genome and pan-genome references facilitates the mining for QR loci and candidate genes (Hurgobin et al. 2018; Alamery et al. 2018; Bayer et al. 2020). In recent years, high throughput markers, including SNPs, have been increasingly used for various applications including genetic mapping, association and genetic diversity analysis. These genome-wide markers have also been explored to predict offspring performance in a population in Genomic Selection (GS) (Meuwissen et al. 2001), significantly decreasing the breeding cycle. This breeding tool offers an enormous potential for improving genetic gain in economically important traits, including blackleg resistance. Although the application of GS in blackleg resistance is still in infancy, several genome prediction simulations have indicated positive gain from this new approach (Fikere et al. 2018; Kumar et al. 2018). As a promising breeding approach, several models have been developed in recent years to improve the efficiency of GS. These include machine and deep learning models, which have been used for the prediction of yield-related traits in maize (Khaki and Wang 2019) and wheat (Ma et al. 2018) and show potential for disease-related traits.

Here, we review the mapping experiments conducted to uncover blackleg QTL based on bi-parental populations and diversity panels. We emphasized the importance of QR and the possible underlying mechanisms based on recent findings. We also highlight improvements in phenotyping methods employed to speed-up blackleg QR assessment. Furthermore, advances in the genomics-aided search for QR and its causative genes are tackled. This information can be used to accelerate the improvement of blackleg resistance in oilseed rape using integrated breeding approaches such as genomic selection.

## Quantitative resistance and its relevance

Quantitative resistance (QR) is defined as a type of plant immunity conditioned by minor-effect genes, resulting in a distribution of phenotypic values that deviate from the classic Mendelian segregation (St.Clair 2010; Pilet-Nayel et al. 2017). Over the years, there has been increased interest in the application of QR in breeding and crop production. QR is conditioned by multiple genes, which imposes less pressure on pathogens to evolve; thus, QR effectiveness could last longer and be less susceptible to resistance breakdown than R-genes. The incorporation of QR in cultivars has also been shown to enhance the effectiveness of R-genes in several crop-pathosystems, including the Brassica-blackleg pathosystem.

For instance, the 5-year field experiment by Brun et al. (2010) demonstrated that QR may prolong the effectiveness of major R-genes. The introgression line, DarmorMX, which carries both major gene (*Rlm6*) and Darmor QR recorded fewer leaf lesions and stem canker compared with lines devoid of QR until the 5^th^ year of the experiment. The study also found that initial avirulence/virulence alleles remained unchanged over the five-year trial in plots planted with lines containing the Darmor background and the inoculum recovered from these lines was significantly fewer compared to non-Darmor lines. This decrease in infection may be attributed to the combined action of R and QR genes against *L. maculans*: the former restricts pathogen colonization in cotyledons during the early disease development, while the latter prevents further pathogen growth in the petiole and stem. These observations were consistent with the follow-up experiment by Delourme et al. (2014) using the same set-up. They concluded that in the presence of QR, R-gene breakdown may be delayed to 5 years. In another study, the introgression of QR in cultivars harbouring known blackleg R-genes *Rlm1*, *Rlm4* and *Rlm7* resulted in a decrease of blackleg symptoms in field conditions. Resistance in cultivars with both QR and R-genes was also found to be more stable across years and environments (Huang et al. 2018).

As shown in these experiments, QR primarily acts to restrict pathogen growth in several plant parts, hence reducing the build-up of virulence/avirulence alleles from one cropping period to the next. However, QR may not ultimately inhibit the diversification of these alleles which may eventually lead to erosion of QR overtime (Delourme et al. 2014). Nevertheless, QR has the potential to sustainably control blackleg by extending the durability of R-genes, so they can be deployed for longer periods, thereby preventing an immediate blackleg epidemic (Brun et al. 2010).

## Mechanisms of QR

The molecular basis of QR is largely unknown, hindering its full exploitation in crop improvement. In contrast to R-gene-mediated resistance, which is sufficiently explained by the two-tier plant immunity model (Jones and Dangl 2006), a growing number of studies suggest that more elaborate mechanisms underlie QR. R-gene-mediated resistance results in distinguishable phenotypic classes (resistant or susceptible) following the molecular interaction of the host plant and pathogen genes. However, QR exhibits a spectrum of phenotypic values between these categories, which the two-tier plant immunity model does not account for (Vasquez et al. 2018). Recently, an alternative model to explain plant immunity that encompasses both R-gene and QR-mediated resistance, the invasion model of plant immunity, has been proposed (Cook et al. 2015). This model describes resistance as the result of the interaction between pathogen invasion patterns (IPs) and the host’s IP-triggered receptors (IPTRs), which continuously evolve to detect IPs, resulting in a quantitative disease resistance output (Cook et al. 2015; French et al. 2016). IPs can be microbe-associated molecular patterns (MAMPs), effectors, or endogenous elicitors such as Damaged Associated Molecular Patterns (DAMPs), which play a wide range of molecular functions, while IPTRs are a convoluted network of plant receptors that dynamically functions to detect IPs.

Understanding the genetic determinism of QR has been conducted through QTL mapping and association analysis. In these studies, several QR loci have been identified; however, as with most quantitative traits, the exact number of genes in these loci is mostly unknown in any pathosystem (Corwin and Kliebenstein 2017). Nevertheless, several genes conditioning QR have been cloned, providing insights into the role of QR in the overall landscape of plant immunity. Subsequently, several hypotheses have been proposed to explain the possible mechanisms controlling QR (French et al. 2016; Poland et al. 2009). For instance, as several diseases follow a spatio-temporal pattern, QR is thought to be likely conditioned by genes involved in plant morphology and development (Raman et al. 2020a). QR is also proposed to be a product of mutation in basal defence genes involved in defence signal transduction following pathogen recognition (Zipfel et al. 2004). In other studies, several QR loci harbour genes that encode intermediary compounds critical for the production of metabolites used to detoxify pathogen virulence products. Hence, QR may be a component of plants’ chemical warfare against pathogens (Kliebenstein et al. 2005; Bednarek 2012). Furthermore, QR genes that colocalize with major R-genes have been proposed as weak forms of R-genes in several plant genomes, including *B. napus* (Raman et al. 2018). Based on these studies, QR is indeed governed by multiple genes and functions in a wide array of mechanisms. Recent advances in ‘omics’ approaches have allowed the dissection of QR at the whole-genome level with some new themes arising to explain QR mechanisms (Neik et al. 2020). These include the involvement of gene regulatory variations in QR expression and how abiotic stress genes may be recruited to perform plant defence functions (gene exaptation).

### Regulatory variation affects QR expression

Regulatory variation influences patterns of gene expression which ultimately affects phenotypic expression (Pai et al. 2015). The effects of such variation have been studied mostly in traits following simple inheritance but with the recent advances in genomic technologies, its role in complex traits, including disease resistance, is now being unravelled (Barco et al. 2019; Tonnessen et al. 2019; Sucher et al. 2020). For example, in rice, promoters of QR genes conferring broad-spectrum defence against *Magnaporthe oryzae*, *Rhizoctonia solani* and *Xanthomonas oryzae* pv. *oryzae* are enriched with *cis*-Regulatory Modules (CRMs). Polymorphisms in these CRMs were also detected between gene promoters of resistant and susceptible cultivars, indicating that these CRMs have functional roles in disease resistance (Tonnessen et al. 2019). In another study, the pan-transcriptomic analysis within the Pentapetalae subclade, which includes representative species *A. thaliana, Phaseolous vulgaris, Ricinus communis, Helianthus annuus* and *Solanun lycopersicum,* all of which exhibit QR against *S. sclerotiorum*, highlighted frequent regulatory variations within QR-responsive genes (Sucher et al. 2020). A large proportion of differentially expressed genes (DEGs) are conserved QR-responsive genes (orthogroups), but only a small portion of these orthogroups contained commonly upregulated (2.01%) and downregulated (3.56%) genes in all the six species. This indicates that while there are conserved QR-responsive genes within these Pentapetalae species, these genes are differentially regulated within species. It is important to note, however, that the number of orthogroups detected in this study may vary if more accessions within each species were included; hence the global and local gene expression profile described in this study may not entirely represent the transcriptional response during *S. sclerotiorum* infection in this subclade.

The involvement of regulatory variation has not been extensively studied in the Brassica-blackleg pathosystem. However, a recent study (Tirnaz et al. 2020) has shown that promoters of defence genes are highly regulated by DNA methylation, resulting in variation in resistance response against blackleg at the seedling stage. A significantly higher number of differentially methylated defence gene promoters was observed in the resistant cultivar, which also tended to be hypermethylated compared with the susceptible cultivar. Furthermore, the study also found that the degree of methylation was extended for a longer period, particularly in the youngest leaves of the resistant cultivar, compared with the susceptible control. This mechanism is proposed to inhibit further pathogen colonization, resulting in decreased stem infection, and may partly explain how seedling resistance contributes to enhancing adult plant resistance. These experiments have demonstrated that regulatory variation and the mechanisms behind it are important features of plant defence, which can be manifested throughout different stages of plant development. Their direct manipulation will provide an alternative strategy to broaden phenotypic diversity for breeding purposes, including that for blackleg resistance.

### Exaptation of gene function as a mechanism for durable resistance

**Fig. 1:**
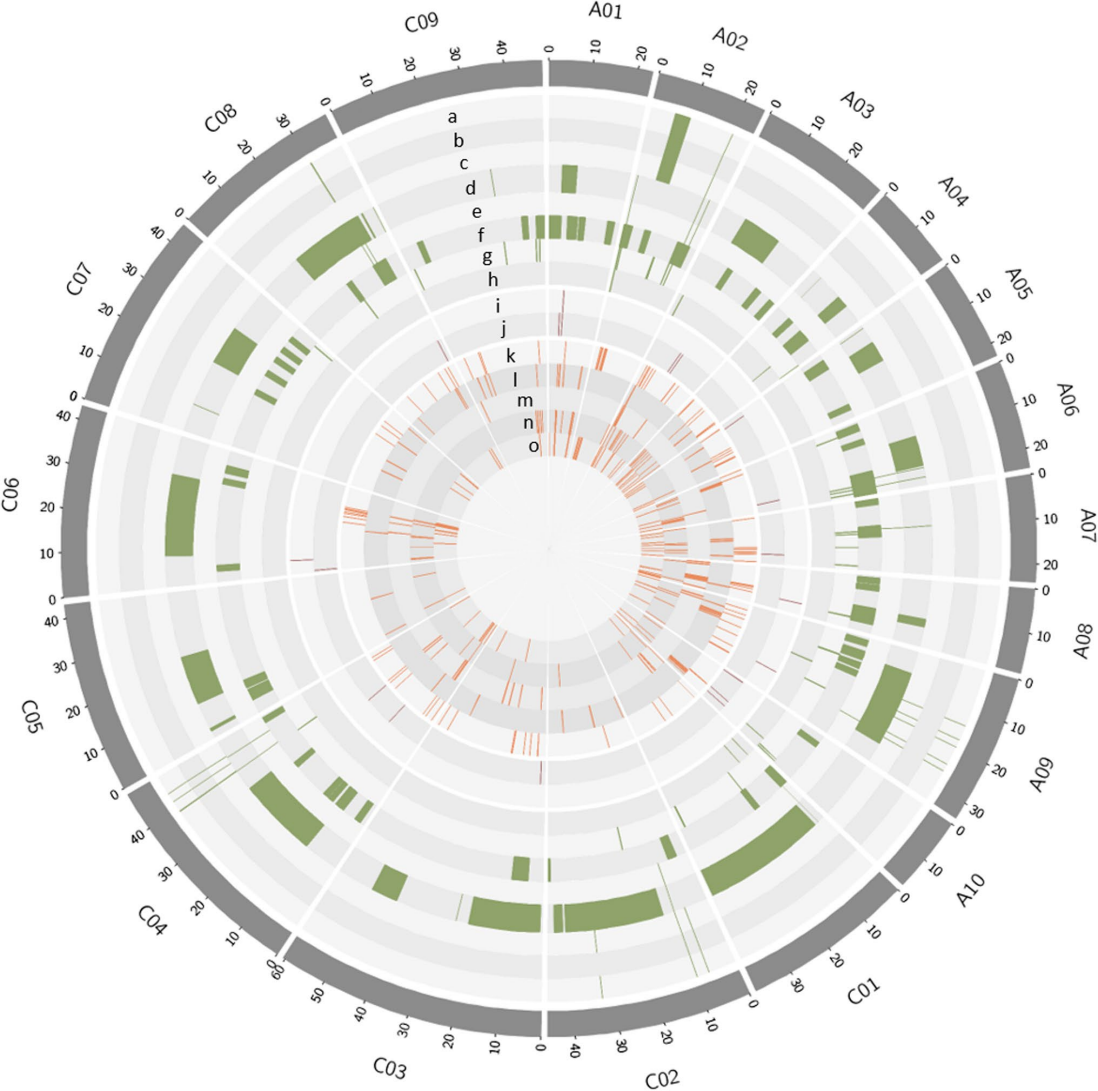
Circos plot displaying the physical position (Mbp) of QTLs and significant genomic regions for blackleg QR in the *Brassica napus* genome (Darmor v4.1). Shown in green highlight are QTL based from Darmor-derived populations (a. Pilet et al. 1998; b. Pilet et al 2001; c. Delourme et al. 2008; d. Jestin et al. 2015; e. Huang et al. 2016; f. Kumar et al. 2018; g. Raman et al. 2018; h. Huang et al. 2019); in red are QTL from non-Darmor populations (i. Larkan et al. 2016; j. Raman et al. 2020a); while in orange are marker positions based from genome-wide association studies (GWAS) (k. Fopa Fomeju et al. 2014; l. Raman et al. 2016; m. Kumar et al. 2018; n. Raman et al. 2020b; o. Fikere et al. 2020a, b). Only studies that disclosed marker sequence information and hence can be mapped to Darmor v4.1 reference genome were included in the diagram

**Fig. 2:**
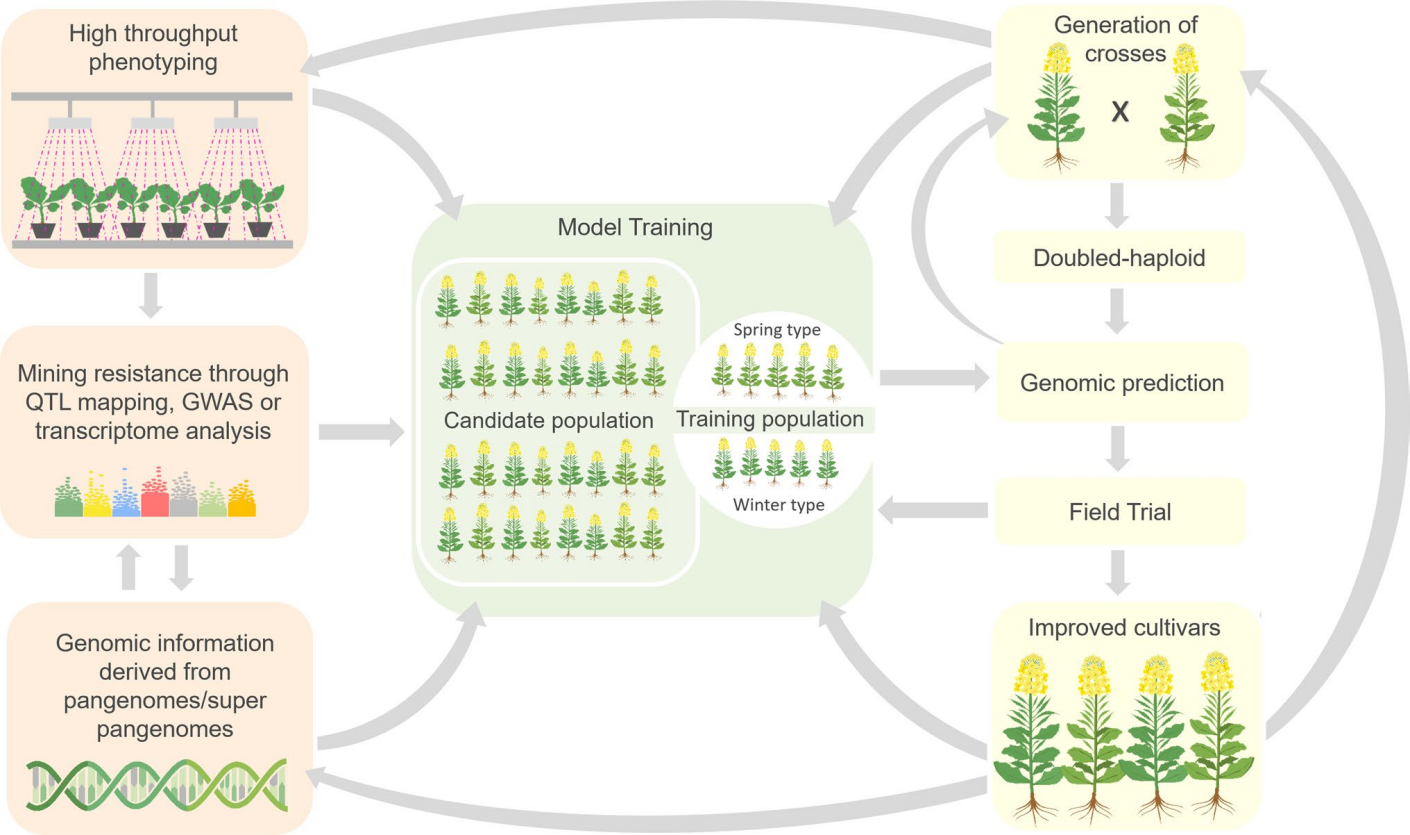
Genomics-guided genetic improvement of blackleg resistance. Genomic selection (GS) which takes advantage of the genome-wide information generated from various platforms, including the improved genome builds and resistance mining efforts such as linkage mapping, GWAS or transcriptome analysis, can be implemented to accelerate the improvement of blackleg resistance in oilseed rape. Information from high throughput phenotyping and genotyping can be integrated with the genome-wide prediction model to account for the overall genetic variance of blackleg resistance. The genomic prediction may also benefit from using separate training populations for winter and spring type oilseed rape. In addition, homozygosity can be achieved at a shorter time by subjecting plants to doubled haploidy (DH) using microspore culture. Furthermore, the model can be updated as new phenotype information is generated. These strategies will increase the selection intensity and decrease the breeding cycle resulting in the accelerated delivery of improved oilseed rape varieties, hence significantly improving genetic gain. Subsequently, improved cultivars may be included as part of the training population or as parents of the subsequent crosses. Genomic information of these improved cultivars can also be added to the pan-genome/super pan-genome which will further enhance the accuracy of genomic prediction in subsequent crosses or breeding populations

During the invasion, pathogens may produce specific molecular cues, such as effectors (Guyon et al. 2014; Haddadi et al. 2019), along with a suite of stress signals (perturbed pH, moisture status and physical strain) that are likewise produced following exposure to abiotic stress (Sucher et al. 2020). Hence, plants that have acclimatized to these abiotic stress signals may also adopt similar mechanisms to resist invading pathogens and the abiotic stress response genes are exapted to function in biotic stress, resulting in a broad-spectrum resistance response. For instance, the abiotic stress response gene ABCG40/PDR12 was found to be induced in several plant species that exhibits QR against *S. sclerotiorum* (Sucher et al. 2020). Because *S. sclerotiorum* emerged much later compared to these plant species, this indicate that ABCG40/PDR12 may have been exapted to function in QR against *S. sclerotiorum* (Sucher et al. 2020). This gene has an ancient role as an abscisic acid importer (Kang et al. 2010) and was proposed to have evolved to function in drought tolerance, seed germination and lateral root formation coincident with land colonization 450 Mya (Campbell et al. 2003; Kang et al. 2010). Previous studies have also implicated this gene in both abiotic and biotic stress conditions, particularly heat stress and upon infection of other fungal pathogens *Alternaria brassicicola and Fusarium oxysporum* (Campbell et al. 2003; Suzuki et al. 2016). In another study, the exaptation of a transposable element (TE) enabled a duplicated iron-stress gene (*CYP82C2*) to function in plant defence biosynthetic pathways (Barco et al. 2019). Crosstalk between biotic and abiotic response pathways has been reported in *Brassica*-pathosystems, including blackleg (Tortosa et al. 2019; Qasim et al. 2020) and it is tempting to speculate that common genes in these pathways might have undergone exaptation to function from one stress condition to another. Gene exaptation presents an interesting view of how QR may have evolved in response to pathogen pressure. Further elucidation of this mechanism may provide additional clues to the origins of QR, which may facilitate identifying causal genes controlling QR for its effective utilization in breeding. Blackleg QR loci are abundant in the *B. napus* genome (Tables 1, 2, 3; Fig. 1, 2), providing a starting point for exploring this mechanism in enhancing blackleg resistance.

**Table 1:** Summary of QTL studies for blackleg QR involving Darmor-derived populations

Population/Genetic cross	Population type	Experimental condition	Phenotype	Marker used	Number of detected QTLs	References
Darmor-*bzh* × Yudal	DH	Field experiment	Plant survival and internal necrosis	RFLP and RAPD	17	Pilet et al. (1998)
Darmor × Samourai	DH	Field experiment	Internal necrosis	Isozyme, RAPD, RFLP	10	Pilet et al. (2001)
Darmor-*bzh* × Yudal	NILs	Field experiment	Internal necrosis	AFLP, SCAR derived RAPD, AFLP or S-SAP	5	Delourme et al. (2008)
Darmor-*bzh x* Yudal	DH	Field experiment	Internal necrosis	SSR,SRAP,SCAR	17	Jestin et al. (2012)
Darmor × Bristol	F_2:3_	Field experiment	Internal necrosis	SSR,SRAP,SCAR	19	Jestin et al. (2015)
Aviso × Bristol	F_2:3_	Field experiment	Internal necrosis	SSR,SRAP,SCAR	11	
Canberra × Bristol	F_2:3_	Field experiment	Internal necrosis	SSR,SRAP,SCAR	15	
Grizzly × Bristol	F_2:3_	Field experiment	Internal necrosis	SSR,SRAP,SCAR	10	
Multi-connected population	meta-analysis	Field experiment (meta-analysis)			13	
Darmor-*bzh x* Yudal	DH	Field experiment (meta-analysis)	Internal necrosis	SCAR derived RAPD, AFLP or S-SAP	17	Huang et al. (2016)
Darmor-*bzh x* Yudal	DH	Field condition (meta-analysis)	Internal necrosis (BLUPs)	SNP	16	Kumar et al. (2018)
Darmor × Samourai	DH			SNP	4	Kumar et al. (2018)
Darmor × Bristol	F_2:3_			SNP	13	Kumar et al. (2018)
Darmor-*bzh* × Yudal	DH	Field and greenhouse (Ascospore shower test)	Plant survival and internal necrosis	DArTseq markers	27	Raman et al. (2018)
Darmor-*bzh* × Yudal	DH	Greenhouse (leaf lamina inoculation)	Lesion width and length	SNP	8	Huang et al. (2019)

*AFLP* amplified fragment length polymorphisms, *DArTseq* diversity array technology sequencing, *RAPD* randomly amplified polymorphic DNA, *RFLP* restriction fragment length polymorphism, *SRAP* sequence-related amplified polymorphism, *SCAR* sequence-characterized amplified regions, *SNP* single nucleotide polymorphism, *S-SAP* sequence-specific amplified polymorphism, *SSR* simple sequence repeat, *DH* doubled-haploid

**Table 2:** Summary of QTL studies for blackleg QR involving non-Darmor-derived populations

Population/Genetic cross	Population type	Experimental condition	Phenotype	Marker used	Number of detected QTLs	References
Major × Stellar	DH	Greenhouse and Field experiment	Internal necrosis	RFLP	9	Ferreira et al. (1995)
Cresor × Westar	DH	Field experiment	Internal necrosis	RFLP	5	Dion et al. (1995)
Caiman × Westar (C_3_W)	DH	Field experiment	Plant survival	EST-SSR and AFLP	3	Kaur et al. (2009)
^AV^Sapphire × Westar (SW);	DH	Field experiment	Plant survival	EST-SSR and AFLP	5	Kaur et al. (2009)
Canberra × Westar (C_4_W)	DH	Field experiment	Plant survival	EST-SSR and AFLP	4	Kaur et al. (2009)
Rainbow × Sapphire (RS)	DH	Field experiment	Plant survival	EST-SSR and AFLP	1	Kaur et al. (2009)
Skipton × AgSpectrum	DH	Greenhouse and Field experiment	Plant survival and Internal necrosis	SSR, SRAP, SCAR	5	Raman et al. (2012a, b)
Topas × AGCastle	DH	Field experiment	Plant survival; internal necrosis	SSR and DArT	16 in single environment analysis; 6 in MET analysis	Larkan et al. (2016)
Topas × AVSapphire	DH	Field experiment	Plant survival; internal necrosis	SSR and DArT	15 in single environment analysis; 6 in MET analysis	Larkan et al. (2016)
RP04 × Ag-Outback	DH	Field and greenhouse (Ascospore shower test)	Plant survival; internal necrosis	DarTseq markers	21	Raman et al. (2020b)

*AFLP* amplified fragment length polymorphisms, *EST-SSR* expressed sequence tags-simple sequence repeat, *DArT* diversity array technology, *RFLP* restriction fragment length polymorphism, *DH* doubled-haploid

**Table 3:** Summary of genome-wide association analysis for Blackleg QR

Diversity panel	Number	Experimental condition	Phenotype	Marker used	Number of significant associations	References
Winter OSR	126	Field experiment	Internal necrosis	SSR and SCAR	23	Jestin et al. (2011)
Winter OSR and Asian spring OSR	116	Field experiment	Internal necrosis	SNP	321 markers corresponding to 64 genomic regions	Fopa Fomeju et al. (2014)
Diverse OSR	179	Glasshouse (Ascospore shower)	Internal necrosis	SNP	600	Raman et al. (2016)
Winter OSR	166	Field experiment (meta-analysis)	Internal necrosis	SNP	18 SNP association with 2006 phenotype data; 22 (2013); 37 (2014); 27 (2015) and 84 with BLUPs	Kumar et al. (2018)
Diverse OSR including *B. napus/Brassica juncea* derivatives, *B. juncea* and *Brassica carinata*	421	Field and greenhouse experiment	Plant survival, Internal necrosis and Upper canopy infection (UPI)	SNP	59 SNP associations	Raman et al. (2020b)
Diverse winter and spring OSR	585	Field experiment	Plant survival and internal necrosis	SNP	674 SNP associations	Fikere et al. (2020a, b)

*SCAR* sequence-characterized amplified regions, *SNP* single nucleotide polymorphisms, *SSR* simple sequence repeat,

## Phenotyping: a challenge for identifying QTL for blackleg resistance

Most blackleg breeding programmes evaluate lines in disease nurseries that have been characterized for consistent disease occurrence (Raman et al. 2020b). However, these disease nurseries can only evaluate a limited number of lines per season. In most cases, large replicated trials are needed to establish genotype stability across different locations and years (Poland and Rutkoski 2016). This approach is labour-intensive and time-consuming, which significantly hampers advancing the genetic gain for blackleg resistance. Several methods have been developed to increase the throughput of quantitative resistance evaluation. The ascospore shower technique (Huang et al. 2006; Marcroft et al. 2012), which is amenable to greenhouse conditions, has been increasingly used in routine QR screening. Another method described is the leaf and petiole inoculation, which evaluates QR in young plants, hence reducing the assessment time (Huang et al. 2014). Advances in digital phenotyping are also becoming attractive methods for high throughput resistance evaluation (Mahlein et al. 2019).

The high sensitivity of QR to genotype by environment interaction (GxE) has been demonstrated by the inconsistent detection of QTL and their variable genetic effects when tested in different environments. For instance, only one out of the 13 QTL for internal infection was consistently detected in a QTL mapping experiment based on a 2-year field evaluation (Raman et al. 2018). The genetic variance displayed by these QTL was also significantly lower compared to a previous experiment utilizing the same genetic population (Kumar et al. 2018). Similarly, only two of the eleven QTL were repeatedly detected across the three field experiments in a recent QTL mapping experiment (Raman et al. 2020b). This is further supported by the different number of significant SNPs detected across sites in multi-environment GWAS experiments (Fikere et al. 2020a, b; Fikere et al. 2018). It was hypothesized that these differences are due to the variable agro-climatic conditions in each site, supporting diverse races of the pathogen. The effectiveness of QR has also been found dependent on temperature: a 10 °C increase in temperature (from 15 to 25 °C) enhanced stem canker severity in a cultivar carrying QR (Huang et al. 2006). This observation is consistent with a previous meta-analysis reporting a higher stem canker severity with increased temperature across Europe (Huang et al. 2016). In the study of Hubbard and Peng (2018), however, increasing the temperature to 32 ºC did not reduce resistance in some QR cultivars, although there is evidence of significant genotype by temperature effects. This result suggests QR-mediated resistance is indeed highly affected by environmental conditions consistent with previous observations. Nevertheless, genetic variability is also present in the *Brassica* gene pool and can be explored for crop improvement.

Other than the confounding effects of GxE interaction, the accuracy of phenotypic evaluation for blackleg resistance has also been dependent on the method of scoring used. Some studies transformed disease scores into infection index, while other studies relied on the per cent survival of plants. Kaur et al (2009) suggested that the use of per cent survival may only be effective in high-disease sites as more QTL are revealed in these environments than those with modest disease levels. In another experiment, the adoption of per cent survival as a resistance metric resulted in a higher narrow-sense heritability (*h*^*2*^) estimate compared with internal infection rating (Larkan et al. 2016). This discrepancy was attributed to the sampling of escape plants for internal infection assessment, which does not represent the population's actual disease severity. It can therefore cause under or overestimation of the QTL effect. Regardless of which scoring system is employed, care should be observed when subjecting plants to field evaluation to avoid mortality due to other factors such as pests and diseases and abiotic stresses, which can significantly affect the assessment.

### The ascospore shower technique

The ascospore shower technique was developed to address some of the limitations of field phenotyping (Huang et al. 2006; Marcroft et al. 2012). Initially, this method was described for evaluating R-gene-mediated resistance but was later modified for screening QR. This method involves suspending an inoculum source, which can either be a culture medium or infected oilseed rape stubbles. These are positioned under a tray cover, which are then placed over plants under evaluation. Spraying of water on the inoculum source triggers the release of ascospores, mimicking a natural infection through a “shower effect” over the plants. After this, inoculated plants are subjected to high humidity for a period to allow disease development. They are then transferred to greenhouse conditions where they are grown until maturity and assessed for internal infection. Assessment of blackleg severity is done by cutting at the crown and inspecting the percentage of discoloration of the crown’s cross-section. This method has been found efficient when the inoculum source is limited, eliminating the need for growing the fungus in culture medium (Huang et al. 2006). The method also allows screening of more lines than field-based evaluation, while decreasing the risk of exposure from various natural factors, including abiotic stress, that can affect disease expression and evaluation.

The reliability of the ascospore shower technique for evaluating blackleg resistance has been effectively demonstrated in R-gene-mediated resistance. For QR, the reliability of the method has been assessed by comparing the heritability estimates of stem canker infection and the consistency of QTL detection relative to field-based approaches. For instance, Raman et al. (2020b) reported an increase in the heritability for stem canker infection from 12% in a field experiment to 61% in an ascospore shower test. Two QTL on chromosomes A01 and A03 were also detected, which were only resolved separately in two field experiments. However, a previous study (Raman et al. 2018) indicated the sensitivity of this method when plants, especially winter oilseed rape types, are subjected to vernalization, as seen in the reduction of heritability estimates in internal infection (44%) compared with field experiments (52 and 71%). This observation confirms the significant effect of changing environmental conditions in the accuracy of assessment, affirming the difficulty of studying QR. Regardless, this approach offers an alternative method for assessing QR.

### Leaf lamina and petiole inoculation

The long period of symptomless *L. maculans* growth in oilseed rape after an infection has been a challenge for assessing QR. Therefore, an ideal way to select for QR is by examining traits that can be readily assessed at the young plant stage, reducing significantly the waiting time for assessment to complete. Huang et al. (2014) described such a method by inoculating the leaf lamina and petioles at 2–3 leaf stages. This method measures the leaf lesion area, the extent of necrosis from the inoculation site and the amount of fungal DNA in the inoculated plants' leaf and petioles. This method could differentiate the resistance response of a non-QR and a QR carrying cultivar, which only takes two to three months to complete. Between inoculum types, ascospores were found to be more effective than pycnidiospores in inducing typical blackleg infection symptoms consistent with previous investigations (Huang et al. 2006; Marcroft et al. 2012). A follow-up mapping experiment was conducted in a DYDH (Darmor/Yudal doubled-haploid) population to test its utility in QR evaluation. Detected QTL were compared with previous mapping experiments conducted in multi-year field trials (Huang et al. 2019). One QTL on chromosome A02 was found coincident with this new experiment and multi-year trials, suggesting that this method can assess some QR components in the field. However, several QTL were unique from this new experiment and previous field experiments. These results indicate that this method might not sufficiently capture all QR loci effects; hence more studies may be needed to verify its effectiveness in screening for QR.

### Digital phenotyping for high throughput disease evaluation

To date, all of the mentioned approaches require subjective technical skill, which often prevents large-scale implementation. Advances in digital phenotyping, including the development of hyperspectral imaging and non-imaging sensors, offer a valuable tool for automatic and high throughput disease assessment at the tissue and whole plant level (Mahlein et al. 2019). Several experiments have demonstrated the potential of such an approach in many pathosystems including barley powdery mildew (Kuska et al. 2015), *Cercospora* *beticola* spots in sugar beet (Leucker et al. 2016), *Septoria* leaf blotch in wheat (Odilbekov et al. 2018), and *Plasmopara viticola* infection in grapes (Oerke et al. 2016). While the current application of digital phenotyping has only been limited to laboratory purposes, efforts are currently underway for its implementation in large-scale field phenotyping. In blackleg, this approach may need rigorous fine-tuning, especially with evaluating stem canker infection due to the need for a destructive assessment. However, the approach can be potentially useful for evaluating seedling resistance since symptoms are expressed in the leaves. Indeed, the development of these phenotyping platforms holds an enormous potential to accelerate crop breeding. However, regardless of which approach is used, the material under evaluation should be certain not to contain any effective R-genes as this might mask the effect of quantitative genes (Van de Wouw and Howlett 2020), resulting in a biased assessment. Therefore, it might be useful to first screen plants for the presence of any R-genes before subjecting them to any approach.

### Upper canopy infection (UCI): an emerging blackleg symptom

The ability of *L. maculans* to infect different plant parts has been highlighted by recent reports describing upper canopy infection (UCI) (Sprague et al. 2018)*.* UCI is a term used to describe infection appearing after plant elongation, specifically in flowers, peduncles, siliques, main stems and branches. This symptom has been linked to changes in agronomic practices such as early sowing and stubble retention in the field. The increasing incidence of UCI in recent years poses another threat to oilseed rape production, which necessitates the need for developing control measures, including the deployment of disease resistance. Initially, it was found that seedling resistance correlates with the reduction of UCI severity in the field; hence, R-gene-based control may be used to manage the symptoms. This observation is supported by the detection of a significant SNP for UCI coincident with the genomic position of *Rlm1* (Raman et al. 2020a). In contrast, there was no correlation between UCI and stem canker severity. As an emerging disease, UCI may need to be targeted for resistance improvement, and phenotyping methods for this should be developed. An ordinal disease severity rating was employed by Raman et al (2020a) to assess UCI as follows: (0) immune reaction (no visible symptoms of UCI); (1) disease present on up to 25% of plant tissue (small lesions, resistant to UCI); (2) disease present on 26–50% of plant tissue; (3) disease present on 51–75% of plant tissue; (4) disease present on > 75% but less than 90% of plant tissue and (5) 100% susceptibility, affecting all tissues, characterized with extensive discoloration, snapping-off vegetative (main and secondary branches) and reproductive structures (siliques). This rating scale may be integrated with the above-mentioned approaches to assess the overall phenotypic response to blackleg. UCI is an ideal candidate for high throughput phenotyping using deep learning systems that have been successfully implemented in a wide variety of crops using hyperspectral and other images (Mahlein et al. 2012; Saleem et al. 2019).

The capacity to generate phenotype data from current crop disease phenotyping approaches significantly lags behind the exponential generation of genomic data and the ability to assemble germplasm from expanding global collaboration among gene banks and scientists. Therefore, the bottleneck for varietal release lies in the ability to generate sizeable phenotypic data rapidly. Indeed, to accelerate breeding, there should be complementation between phenotyping and genotyping platforms in terms of efficiency and throughput. This scenario could be realized for blackleg resistance (Fig. 2) and *Brassica* breeding in general as there are ongoing efforts to enrich current genomic resources through the construction of pan-genomes and the development of image-based high throughput phenotyping methods.

## Genetics of quantitative resistance to blackleg

While studies on the *Brassica-L. maculans* interaction have long been pursued (Sjodin & Glimelius, 1988), it was only since the availability of DNA markers that thorough genetic investigations have been carried out. These studies have established the complex genetic control of blackleg resistance. Early linkage and QTL mapping using bi-parental crosses for mining blackleg resistance loci were conducted using low-throughput markers including restriction fragment length polymorphism (RLFP) and random amplified polymorphic DNA (RAPD) markers (Dion et al. 1995; Pilet et al. 1998, 2001). The amenability of *B. napus* to microspore culture has also made it possible to generate doubled-haploid (DH) plants on which most of these mapping studies were conducted. DH populations are genetically uniform, allowing replicated evaluation in multiple environments (Pink et al. 2008). In some instances, F_2_ populations were used as they allow estimation of the dominance effects of QTL, which could be valuable in hybrid breeding (Pilet et al. 2001). Near-isogenic lines (NILs) are also produced to validate the effects of blackleg QTL (Delourme et al. 2008). In recent years, diversity panels have been employed for determining resistance trait–marker association through GWAS. This bypasses the need to generate crosses for mapping population development, saving considerable time to search for causative genomic regions conditioning QR. In this section, we summarize some of the major accomplishments in mining for blackleg QR using bi-parental mapping populations and diversity panels (Tables 1, 2, 3; Fig. 1).

### Darmor is a primary source of quantitative resistance against blackleg

Several QTL analyses have been conducted to discover and/or confirm the presence of QR alleles in Darmor. Four genomic regions controlling field plant survival (P) and stem canker severity expressed as disease index (I) were located in linkage groups (LGs) 2 (C02), 3 (C04), 5(A09) and 11(A02) of the Darmor/Yudal (DY) genetic map (Pilet et al. 1998). The stability of these QTLs was further assessed in a follow-up experiment using Darmor/Samourai (DS) DH and F_2:3_ mapping populations (Pilet et al. 2001). Comparative analysis in the two linkage maps found four common QTLs to both populations located on chromosomes A02, C02, C04 and C08. These QTL were also detected in an experiment by Jestin et al. (2012) confirming the consistency of these Darmor QTL. The analysis of the F_2:3_ population, on the other hand, revealed dominant control of field resistance for QTL located on A02 of the DS genetic map. In the study of Delourme et al. (2008), the introgression of Darmor QTL *Lm02* (A02) and *Lm09* (A09) into the susceptible cultivar Yudal resulted in significantly lower stem canker infection in near-isogenic lines (NILs). In contrast, NILs carrying *LmC2.1* and *LmC2.2* (C02) and *LmC4.2* (C04) QTLs exhibited severe stem canker infection comparable to the susceptible checks and hence were considered not effective for reducing blackleg at the mature stage.

The increasing number of genetic mapping studies employing Darmor-derived populations has provided researchers the opportunity to integrate previous and recent sets of phenotypic data to analyse the stability of Darmor-derived resistance. Besides, the availability of genome builds for *B. napus* (Chalhoub et al. 2014) and robust marker systems have confirmed the durability of Darmor alleles, and in general, provided insights into the organization of blackleg QR loci in oilseed rape. A multiple-connected population derived from crossing Darmor and three other resistant cultivars (Aviso, Canberra and Grizzly) to a susceptible cultivar, Bristol (DB), was used to compare the effects of different resistance alleles and to validate the presence of Darmor alleles (Jestin et al. 2015). The study detected Darmor QTL spanning chromosomes A01, A02, A04, A06, A08, C01, C02, C04, C05 and C08, with a few of these regions also co-located with other resistance sources, providing evidence of the introgression of Darmor alleles in several cultivars. The meta-analysis of phenotype data obtained from the experiments of Pilet et al.(1998) (1995–1996 data); Jestin et al. (2012); and (Huang et al. 2016) 2008–2009 Rothamsted, UK, experiment revealed consensus QTL of importance for the European region. The study detected a total of 17 field resistance genomic regions, of which four regions (A02, C02, C04 and C08) coincided with the genomic position in the Pilet et al. (1998) experiment, while six new QTLs were also identified. Considering the five-year data, six QTLs located on chromosomes A02, A07, A08, C02, C04 and C08 were found less sensitive to environmental or year effects and were considered stable QTLs for the European growing conditions. In a further analysis, best linear unbiased prediction (BLUP) estimates were generated for phenotypic data obtained from previous experiments (Huang et al. 2016; Jestin et al. 2012, 2015; Pilet et al. 2001) and integrated with genotype information from Illumina SNP arrays (8, 20 and 60 K) to map common blackleg resistance QTL (Kumar et al. 2018). This new analysis resulted in the identification of at least 16 common genomic regions underlying QR in the three Darmor-based populations (DY, DS and DB). Of these, one region located on chromosome C04 had overlapping intervals between the different populations; fifteen were common in at least two populations, wherein most (9) of these common regions were shared between DY and DB populations. Furthermore, nine regions were common to previously reported QTL in the connected multi-parental design (Jestin et al. 2015), while few regions reside near the significant SNPs of the GWAS study of Raman et al. (2016).

The global importance of Darmor-derived resistance was also highlighted with the detection of several QTLs in Australian field conditions (Raman et al. 2018). The multi-year evaluation using an ascospore shower test and field phenotyping enabled the identification of 27 QTL regions associated with blackleg resistance in the DY population. Of these, seven regions located on chromosomes A02, A07, A09, A10, C01 and C09 were consistently detected across experiments, and eight loci were found to be common among France, UK and Australian conditions. As Darmor is a European cultivar, this implied that Darmor QR is also expressed and effective in Australian conditions. The consistent detection of Darmor QTL in these studies emphasized the importance of Darmor and its parent Jet Neuf as key sources of QR against blackleg, which have persisted through several generations since they were first used in breeding programmes. On the other hand, the non-detection of some of these genomic from one experiment to another reflects the sensitivity of quantitative mediated resistance. Nevertheless, consistent Darmor genomic regions may be further interrogated for candidate genes controlling QR and may aid in the development of robust markers for MAS.

### QTL in germplasm other than Darmor

The prevalence of blackleg through the years has encouraged a continuous search for durable blackleg resistance in germplasm other than Darmor. The pioneering work of Ferreira et al. (1995) resulted in the identification of major and minor-effect QTL controlling seedling and adult plant blackleg resistance, respectively, in the Major/Stellar DH mapping population. Two field resistance loci located on LGs 12 and 2 were mapped together with the major seedling resistance locus, *LEM 1* on LG 6. The major locus, *LmFr1*, from the cultivar Cresor, was thought to control adult plant resistance as it was first mapped in field screening conditions (Dion et al. 1995); however, subsequent investigations classified it as one of the R-genes located on chromosome A07 of *B. napus*. Kaur et al. (2009) mapped several QTLs from four DH mapping populations derived from four resistant sources; Caiman, Canberra, ^AV^Sapphire and Rainbow. Caiman, Canberra and ^AV^Sapphire were all crossed to Westar (susceptible) to create the first three mapping populations, while the fourth population was derived from an ^AV^Sapphire/Rainbow cross. Composite interval mapping (CIM) detected one QTL on chromosome C05 of the Caiman/Westar cross, and chromosomes A01/C01 of the Canberra/Westar cross, while multiple QTLs were mapped on chromosomes A01, C01 and LG 1 from the ^AV^Sapphire/Westar DH population. In contrast, no significant QTLs were detected in the Rainbow/^AV^Sapphire cross, which might be since they are both resistant cultivars, and there is no segregation of resistance alleles. The A01 QTL was suggested to be the same QTL identified in the Skipton/AgSpectrum DH population, designated as *QRlm.wwai-A1a*. This region, together with another QTL located on chromosome A10, designated *QRlm.wwai-A10,* showed consistent effects at both the cotyledon and adult plant stages (Raman et al. 2012a, b). Another QTL on chromosome A10 from Caiman/Westar and Canberra/Westar cross was identified using simple interval mapping (SIM) as a detection method. Further comparative analysis of this region revealed genomic coincidence with major genes *LepR2* and *LepR3* in *B. rapa ssp. sylvestris* (Rimmer 2006). Similarly, one QTL on chromosome A02 of the ^AV^Sapphire/Westar cross was also found to colocalize with the previously mapped major gene *LepR1* (Yu et al. 2008).

Other Australian *B. napus* cultivars known to display QR were also explored as parents for mapping QR loci. For example, AG-Castle and AG-Sapphire were crossed to a common susceptible parent (Topas) to create the DH mapping populations designated as TC (Topas/AG-Castle) and TS (Topas/AG-Sapphire) (Larkan et al. 2016). Four highly heritable and consistent QTLs were identified on chromosomes A01, A09, A08 and C06, and these genomic locations coincided with previous QTL for blackleg QR. The genomic interval of TC QTL on chromosome A01 is consistent with the reported QTL from the study of Raman et al. (2012b) and Jestin et al. (2015), and the TC A08 QTL colocalized with QTL mapped in the DB and DY populations (Delourme et al. 2008; Huang et al. 2016; Jestin et al. 2015), while the TS chromosome A09 QTL co-located with the *LmA09* interval from the DY map, which was also previously reported to correspond to a syntenic *A. thaliana* region (Delourme et al. 2008). The chromosome C06 QTL found in both TC and TS populations corresponded to the genomic region in Aviso and DB population, spanning several candidate resistance genes (Jestin et al. 2015). Further analysis of the TC A01 peak genomic interval revealed a cluster of cysteine-rich receptor-like kinase genes (CRKs), which have been implicated with disease resistance in several plant-pathogen interactions, suggesting these genes may play a role in QR.

The recent multi-environment QTL mapping by Raman et al. (2020b), using a DH population from the cross between two Australian varieties RP04 and Ag-Outback, also detected QTL in previously described genomic regions including on chromosomes A01, A03, A05, A06, A07, A10, C03, C04, C06 and C08 (Fikere et al. 2020a, b; Jestin et al. 2012, 2015; Kumar et al. 2018; Larkan et al. 2016; Raman et al. 2016; Raman et al. 2012a, b). The QTL on A07, which was detected using a cotyledon shower test localized within marker intervals of major genes *Rlm3* and *Rlm4*. However, these genes were not detected in the field evaluation and are therefore not effective in field conditions, consistent with previous observations (Marcroft et al. 2012). Sequence analysis of significant markers linked with consistent QTL on chromosomes A03, A07 and C03 revealed high similarities with genes exhibiting disease resistance features (Mayerhofer et al. 2005; Curto et al. 2015) and can be considered candidate genes for these QTLs. The genomic coincidence of loci between Australian and French populations demonstrates a selection of durable, however genetically narrow QR loci, further suggesting a need to broaden the genetic base for QR.

The re-synthesis of *B. napus* cultivars using wild accessions of *B. rapa* subsp. *sylvestris* as a source of the A genome resulted in breeding lines expressing a high level of seedling and adult plant resistance. These include lines AD9, AD49 and MC1-8 400, which through genetic mapping were found to harbour sylvestris-derived resistance genes *LepR1*, *LepR2* and both *LepR1* and *LepR2*, respectively (Li and Cowling 2003; Yu et al. 2005, 2008). Another line, Surpass 400, which was released as a variety in Australia in 2001, was initially very effective at providing resistance at both seedling and adult plant stages, but became susceptible within just three years of its release. Li and Cowling (2003) first reported a single dominant allele controlling Surpass 400 resistance. This was subsequently located on chromosome A10 and designated as *LepR3* (Yu et al. 2008). Both *LepR3* and *Rlm1* interact with the same *AvrLm1* gene. However, the cloning experiment by Larkan et al. (2013) revealed that *LepR3* is allelic to another major gene, *Rlm2*, while the specific gene controlling *Rlm1* is yet to be confirmed. In a separate study by Long et al. (2011), two independent loci, designated as *BLMR1* and *BLMR2* were identified in Surpass 400, flanking the previously mapped *LepR3* locus (Yu et al. 2008). NILs carrying the *BLMR1* locus exhibited a consistent resistant phenotype when infected with *L. maculans* at the cotyledon stage; this gene was later shown to be the same *LepR3* gene through cloning (Zhou et al. 2019a, b, c). On the other hand, the evaluation of NILs carrying the *BLRM2* locus found a strong correlation between the intermediate resistance at the cotyledon stage with adult plant resistance, suggesting *BLRM2* mediates quantitative resistance (Dandena et al. 2019). Genomic analysis of this locus will provide information on its gene content and may be explored in validation experiments. This will aid in unravelling the mechanisms underpinning QR variation derived from the introgression of wild *Brassica* species.

### Genome-wide association studies to discover QR

There is a significant time-lag from QTL discovery to its application in crop improvement (Snowdon and Friedt 2004; Ersoz et al. 2007); however, this limitation can be overcome using methods such as genome-wide association studies (GWAS) that take advantage of the extensive recombination events captured in natural populations. In several instances, GWAS has been implemented as an effective complementary approach to confirm and validate QTL detected with bi-parental QTL mapping.

One of the first GWAS for blackleg resistance was described by Jestin et al. (2011) to confirm QTL previously mapped in Darmor and discover new alleles associated with adult plant resistance. A *B. napus* panel composed of 128 lines exhibiting diverse responses to blackleg was phenotyped and genotyped using 72 SSR markers. The association analysis detected 23 significant alleles; twelve of which were positioned on the DY map. Among these, seven were located near a previous QTL interval detected in Pilet et al. (1998) and Delourme et al. (2008). The other five alleles were located in regions not previously mapped to known QTL and were considered new alleles. These included QTL markers described by Jestin et al. (2015) in their multi-connected population study. The eleven other significant alleles were not found in the previous DY linkage map, which may represent novel alleles. The GWA study of Fopa Fomeju et al. (2014) found several genomic regions controlling quantitative resistance in a panel of 116 OSR which were located in strictly duplicated homeologous regions of the *B. napus* genome. These duplicated regions were located in six of the 24 conserved collinear blocks between *A. thaliana* and *B. napus*. Further investigation uncovered that these duplicated regions contain some of the QTLs detected in previous linkage mapping experiments (Pilet et al. 1998). Findings of this study supported the involvement of duplicated regions in controlling important traits including disease resistance in polyploid plant species. A follow-up comparative genomic analysis revealed genes involved in stress response in these regions, which may control QR (Fopa Fomeju et al. 2015). Further expression analysis of these genes will confirm the involvement of duplicated regions and genes in QR expression.

In another GWAS, Raman et al. (2016) identified significant marker–trait associations representing both known and unknown loci in the GWAS panel composed of 179 *B. napus* accessions. The significant associations were further inspected using linkage analyses, enabling the validation of known R-genes and identification of a new gene conferring adult plant resistance, designated as *Rlm12*. This gene was located on chromosome A01, consistent with the genomic location reported in previous linkage mapping experiments, including that of Raman et al. (2012b) who used the same mapping population (Skipton/AgSpectrum). However, since the corresponding *Avr *gene (*AvrLm12*) has not been identified to date, the identity of this new locus remains to be established. In another study (Kumar et al. 2018), a multi-year association analysis using a panel of 166 winter-type OSR devoid of any known R-genes enabled the identification of GWAS-BLUP regions for adult plant resistance. Some were homologous with the detected regions in the complementary QTL mapping experiment ("Darmor is a primary source of quantitative resistance against blackleg" section). In particular, a significant GWAS peak on chromosome A08 overlapped with a QTL region in the DY population, while another significant association on C04 was found colocalized with a QTL region in DB and DS populations. Together with other minor-effect associations, these regions coincided with the genomic regions reported by Fopa Fomeju et al (2014).

In a recent experiment, 421 diverse accessions of *Brassica* species, including 395 *B. napus*, 21 *B. napus/Brassica juncea* derivatives, one *B. juncea* and four *Brassica carinata* were subjected to GWAS analysis to identify loci controlling QR (Raman et al. 2020a). Other than the usual phenotype metrics (plant survival and internal infection), the study also assessed the emerging blackleg symptom of upper canopy infection (UCI) to determine its genetic control. The study found a total of 59 significant SNP associations across 17 chromosomes of *B. napus*, with most of these SNPs found colocalizing with previous QR-associated genomic regions (Fikere et al. 2018, 2020a, b; Fopa Fomeju et al. 2014; Jestin et al. 2011; Kumar et al. 2018; Larkan et al. 2016; Rahman et al. 2016; Raman et al. 2020a). Based on the SNP markers' physical position, chromosomes A04 and A07 were identified as genomic hotspots for blackleg resistance. These two chromosomes also contain significant associations for loci controlling UCI. In another experiment, a whole-genome sequence-GWAS (WGS-GWAS) meta-analysis in 585 diverse winter and spring type OSR found 79 genomic regions harbouring 674 SNPs controlling blackleg resistance traits including per cent survival and average internal infection (Fikere et al. 2020a, b). Twenty-seven of these regions colocalized with known major blackleg R-genes, while 52 were deduced as new regions. A few of these regions coincide with the genomic positions of QTLs and significant markers found in other studies (Raman et al. 2016, 2020a, b) (Fig. 1). The colocalization of GWAS peaks in these experiments with previous and currently mapped QTL regions suggests that GWAS and linkage mapping are efficient complementary approaches for detecting QTL.

## Genomics-aided identification of QR loci and genes

Genomic resources for *Brassicas* are rapidly expanding since the sequencing of the first *Brassica* species *(B. rapa; *AA) was initiated by the Multinational *Brassica* Genome Project (Wang et al., 2011). At present, reference genomes for other major species, *B. carinata* (Song et al. 2021), *B. oleracea* (Liu et al. 2014; Parkin et al. 2014; Belser et al. 2018)), *B. nigra*, *B. juncea* (Yang et al. 2016) and *B. napus* (Chalhoub et al. 2014) are currently available and progressively being enriched as new information are generated from the accelerated development of sequencing platforms and improvement in computational analyses. Recent efforts have concentrated on the construction of pan-genomes, representing the overall genomic repertoire of a species, overcoming the limitation of using single genome reference (Bayer et al. 2020; Golicz et al. 2016a, b; Hurgobin and Edwards 2017). These efforts provide platforms to accelerate the identification of genes underlying agronomically important traits, including disease resistance and the discovery of high-quality diagnostic molecular markers for breeding. Complementing this rapid build-up of genomic information is the establishment of information resources in the form of genome and pan-genome browsers, databases and several bioinformatics pipelines, most of which have been made available publicly.

### Genome-wide markers for characterizing QR loci and genes

Genome-wide markers, including SNPs, obtained from whole-genome resequencing, genotype-by-sequencing and SNP arrays, have proven useful for identifying loci controlling both qualitative and quantitative resistance against blackleg. These markers are physically linked to annotated genes in the reference genomes. Therefore, they can be used to identify putative candidate genes that could then be targeted for validation experiments using transgenic or genome-editing approaches.

*Brassica* SNP arrays (60 K, 20 K and 8 K) (Chalhoub et al. 2014; Clarke et al. 2016; Delourme et al. 2013) were employed in a GWAS experiment that mapped significant regions containing several stress-responsive genes, suspected to play a role in QR (Kumar et al. 2018). At least 6.2% of the genes exhibited stress response features, including a mitogen-activated protein kinase 16 (MAPK16) and a transcription factor (WRKY), which were found surrounding the significant associations on chromosome A08. On chromosome A09, the most significant SNP was found to localize with a MAPK7, while the LD region of C01 harbours two defensin-like proteins. Additionally, a QTL mapping study (Raman et al. 2020b) that utilized DarTseq markers found a high similarity between the significant markers with stress-responsive *A. thaliana* genes, including a JUMONJI transcription factor, protein kinase and helicase domain-containing protein. All of these genes have been known to play a role in several cellular processes such as signalling and transcriptional regulation during stress (Lay and Anderson 2005; Haddadi et al. 2016). In a recent GWAS experiment using 12,414 high-quality SNPs, at least 59 SNP associations with blackleg QR, including loci for UCI, covering 17 *B. napus* chromosomes, were detected (Raman et al. 2020a). Inspection of the physical coincidence of these markers with annotated genes in the reference genome revealed the possible involvement of genes involved in both pathogen-associated molecular pattern (PAMP)-triggered immunity (PTI) and effector-triggered immunity (ETI) mediated response, indicating that QR-mediated resistance also shares some defence-response characteristics with R-genes.

However, it remains a significant challenge to identify QR genes using *in-silico* approaches due to the lack of identifiable features such as those described for R-genes. On the other hand, issues may also arise in R-gene prediction due to the differences in approaches employed during gene annotation, such as repeat masking (Bayer et al. 2018). This may have serious consequences in mining for resistance genes in that other genes may be falsely classified as resistance genes or novel resistance genes may not be detected; hence rigorous re-evaluation of annotation pipelines has been highly encouraged. Nevertheless, advances in genomic resources have facilitated the identification of candidate genes and markers, including SNPs to study the mechanisms involved in blackleg resistance. Aside from genetic mapping, SNP arrays also provide tools for studying the genetic relationship of *Brassica* species displaying variability in blackleg resistance and inferring the resistance introgression patterns in breeding lines.

### Transcriptomics for dissecting QR mechanisms

The interrogation of the mechanisms underlying disease resistance is mostly conducted using gene expression analysis. The availability of genomic resources that allow direct quantification of genes through sequencing (RNA sequencing, RNAseq), enables a genome-wide investigation of genes involved in host defence (Metzker 2010; Westermann et al. 2012; Weber 2015). In most transcriptome studies, the typical blackleg R-gene-mediated resistance has been characterized by the expression of genes for hormone signalling, cell wall thickening, chitin, glucosinolate production and induction of pathogenesis-related proteins following the interaction of *Rlm* genes with their specific *Avr* *L. maculans* counterpart (Bednarek 2012; Chen et al. 2016; Haddadi et al. 2016; Zhou et al. 2019a, b, c). For QR, the role of a diverse set of genes has been proposed. In a recent transcriptome profiling experiment, Hubbard et al. (2020) found genes controlling programmed cell death (PCD), reactive oxygen species (ROS) and intracellular endomembrane transport as critical regulators of QR. The ROS burst was coincident with inhibited fungal growth and rapid cell death. Hence, it was proposed that a possible QR mechanism is by limiting *L. maculans* colonization through ROS-mediated rapid cell death. However, it is important to note that this study was conducted at the cotyledon stage based on findings that correlated cotyledon resistance with reduced canker development at the adult stage (Hubbard and Peng 2018). This result contrast with previous reports indicating resistance at the cotyledon stage does not always result in resistance at the adult plant stage (Raman et al. 2016, 2018). As QR has been mostly described at the adult plant stage, a follow-up transcriptome study is needed to confirm whether such a mechanism is also active at the maturity stage.

Developmental genes were also proposed to partially condition QR, as evidenced by the colocalization of a major QR loci with a genomic region controlling maturity and dwarfness on chromosome A06 (Raman et al. 2016, 2018). In other *Brassica* diseases for which resistance is primarily based on QR, including stem rot and black rot, several genes were found to contribute to QR. These include genes functioning in hormone synthesis, production of secondary metabolites, regulation of transcription factors, several metabolic pathways (Qasim et al. 2020) and calcium signalling (Tortosa et al. 2019). Given the complexity of the mechanisms described in these studies, it may be helpful to adopt a systems genetics approach integrating transcriptome data and association genetics to identify transcriptional networks controlling QR (Christie et al. 2017). This approach will help navigate the identification of candidate genes within these complex networks allowing a more targeted approach in molecular marker development, which can be integrated into breeding (i.e. genomic selection, Sect. 6.4).

### Implication of structural variations (SVs) in QR

The exponential increase in genomic information brought by the rapid development in sequencing has led to the discovery of another type of variation called structural variation (SV) (Gabur et al. 2019). The two most recognized forms of SVs are copy number (CNV) and presence/absence variation (PAV); however, recently, homologous exchanges (HEs) were also proposed as another type of SV (Schiessl et al. 2019). These genomic variations affect the gene content of individuals of a species and therefore influence trait variability and expression. For instance, the QR locus *Rgh1* against the cyst nematode has been linked to the presence of multiple copies of three putative genes at the locus (Cook et al. 2012). Several duplicated genes were also found in genomic regions affecting blackleg QR (Fopa Fomeju et al. 2015). Likewise, 23–51% of the genes residing in QTL conferring resistance against *V. longisporum, *which causes stem-striping disease in *B. napus* (Gabur et al. 2020) have been shown to be influenced by gene PAVs. These studies demonstrate the role of SVs in disease resistance, and as more accessions are sequenced, its involvement in driving phenotypic diversity will likely become better understood.

The discovery of SVs has made it apparent that a single reference genome cannot represent the entire genomic repertoire of a species and hence may be considered as already an obsolete genomic resource (Hurgobin and Edwards 2017; Bayer et al. 2020; Danilevicz et al. 2020; Golicz et al. 2020). To address this, pan-genomes, which represent the complete and non-redundant set of genes in the species are being actively pursued. A pan-genome can identify which genes are considered as core genes, present in all members of a species, and which are variable or dispensable and only present in some individuals (Tettelin et al. 2005). For instance, the *B. oleracea* pan-genome spanning 587 Mbp was found to contain 81.3% core gene models, and the remaining (18.7%) are categorized as variable genes (Golicz et al. 2016a, b). Variable genes are usually enriched with disease resistance genes (Tian et al. 2003) and disease resistance genes are abundant in the variable genome of diverse species (Bayer et al. 2019; Montenegro et al. 2017; Yu et al. 2019; Zhao et al. 2020). In another pan-genomic analysis, 1749 resistance gene analogs (RGAs) were detected across 50 synthetic and non-synthetic *B. napus* (Dolatabadian et al. 2020). Of these, 368 RGAs were absent from the Darmor-*bzh* reference genome, with some 106 RGA candidates linked to blackleg resistance QTL. The large proportion of undetected RGAs may contain important genes that were possibly missed out in previous comparative mapping and genetic analysis and would have been useful for breeding. Indeed, the incorporation of more sequence information from different cultivars may increase the genomic information in pan-genomes. A proposal to include genome information from wild species termed as super pan-genome will further incorporate more diversity encompassing the genus-level pan-genomes (Khan et al. 2020). This wealth of genomic data is expected to provide insights into the genomic organization of *Brassica* species, which will help accelerate the identification of genes, including QR genes for blackleg.

### Genomic selection: potential for improving blackleg resistance

Genomic selection (GS), as originally proposed by Meuwissen et al. (2001), is a form of MAS, wherein genome-wide marker information is used to predict the genetic merit of individuals in a population. This approach involves generating a model (GS model) based on the relationship between the genome-wide marker data and phenotype information in a training population. The trained model is then applied to a candidate population to predict the breeding values (genome estimated breeding value, GEBV) of individuals, using only their genotype information (Meuwissen et al. 2001; Spindel et al. 2015). Several statistical approaches, including deep learning methods (Zingaretti et al. 2020), have been developed for GS in recent years; however, only a small number of papers have investigated the performance of these models which show the potential to outperform traditional GS tools (Ma et al. 2018; Montesinos-López et al. 2018a, b; Khaki and Wang 2019). Regardless, these approaches have the potential to accurately predict offspring performance enabling early-generation and off-season selection, which significantly shortens the breeding cycle. Furthermore, more individuals can be evaluated at a given time and ensure that only the most ideal individuals, based on their GEVBs are advanced to the next level stage of evaluation. These advantages altogether contribute to increasing the rate of genetic gain for a particular trait, making GS a powerful breeding tool.

GS can be particularly beneficial for improving traits displaying complex genetic architecture such as disease resistance, which has been known to have qualitative and quantitative genetic components in most plant-pathogen interactions. GS models can capture both genetic effects to predict resistance phenotype, making disease resistance improvement a good target for GS approaches (Poland and Rutkoski 2016). The accuracy of genome prediction models has been evaluated for a range of diseases including wheat rust (Rutkoski et al. 2015a, b, 2014), *Fusarium* head blight of barley (Lorenz et al. 2012), flax pasmo (He et al. 2019), witches’ broom disease and frosty pod rot disease of cacao (McElroy et al. 2018), cassava brown streak disease (Kayondo et al. 2018), ryegrass crown rot (Arojju et al. 2018) and maize ear rot (dos Santos et al. 2016). These studies highlighted the potential of GS approaches in improving disease resistance in these crops.

Previously, genome-wide models have been explored for the prediction of various traits in oilseed rape, including agronomic (Wurschum et al. 2014) and seed quality traits (Werner et al. 2018), and test-cross performance for hybrid breeding (Jan et al. 2016). Fikere et al. (2018) and Kumar et al. (2018) described some pioneering work on the applicability of genome-wide models in predicting blackleg resistance in oilseed rape. Fikere et al. (2018) used three different genome-wide prediction models with 98,054 SNPs to predict seedling emergence, survival rate and internal infection in spring- and winter-type oilseed rape plants evaluated under two disease nurseries in Australia. Their prediction models obtained a moderate to high prediction accuracy ranging from 0.35 to 0.74. Different accuracy values were also obtained when the GS model was strictly implemented in each oilseed rape type with 0.30–0.69 in the spring set and from 0.19 to 0.71 within the winter set. In the study of Kumar et al. (2018), prediction of blackleg resistance in *B. napus* using different combinations of training sets and SNP markers yielded a variable prediction accuracy from 20 to 70%. Using a 60:40% ratio for training and validation sets, the prediction ability of their model ranged from 0.68 to 0.88 with different SNP marker densities. Furthermore, integrating the year-wise blackleg disease index yielded a prediction accuracy of 0.80 for 2014 and 2015 and 0.81 for 2013.

While genomic prediction (GP) models are originally designed to capture minor-effect loci, more advanced algorithms can incorporate prior biological information such as major effect genes or regulatory regions fitted as fixed effects in several models. These include rrBLUP, and Bayesian models, BayesA, BayesB, BayesCπ, Bayesan R, Bayesan RC and Bayesian LASSO (Heslot et al. 2012). Other models account for non-additive effects in estimating the total genetic variance, such as Reproducing Kernel Hilbert Space (RKHS) (Gianola and Van Kaam 2008) and Random Forest (RF) (Ogutu et al. 2011). The differences of these models are discussed in several reviews (Crossa et al. 2017; Poland and Rutkoski 2016), but in essence, all of them can take into account both minor- and major-effect QTLs, reflecting a more accurate estimate of the overall genetic variance. Other machine and deep learning models also have potential applications in genomic prediction, due to their ability to capture complex nonlinear relationships, and these models are gaining use in plants (Ma et al. 2018; Khaki and Wang 2019) and the medical sciences (Zhou et al. 2019a, b, c). Blackleg resistance improvement can leverage on these models by incorporating previous R-gene and QTL information. The integration of prior blackleg resistance QTL information using Bayesan RC, improved the accuracy of prediction in winter and spring type oilseed rape (Fikere et al. 2018). More importantly, it was found that only less than 30% of the genetic variance for blackleg resistance has been captured in present cultivars, which means that a large proportion of this genetic variance remains to be uncovered. Furthermore, GP is particularly advantageous in the case of blackleg, wherein some of the known R-genes have been shown to mask the effect of other major and minor genes. Applying GS will circumvent this problem as QR genes will still be selected regardless of whether an R-gene is present or not; however, one caveat for its effective implementation is to ensure that the training population used is devoid of any effective R-gene (Poland and Rutkoski 2016).

The accuracy of genomic prediction is dependent on many factors including trait heritability, linkage LD decay, marker density and size of the training population (Daetwyler et al. 2010). High trait heritability, although making GS more accurate, is counterproductive compared with phenotypic selection based on per cycle and per-unit genetic gain (Heffner et al. 2010). In this case, GP will only be advantageous over phenotypic selection when the intensity of selection is increased, coupled with the reduction of the breeding cycle (Poland and Rutkoski 2016). With blackleg resistance, several authors have reported variable heritability values depending on the experimental condition. In a recent study by Raman et al. (2020b), the average broad-sense heritability for QR in field condition was 45%, while using the ascospore shower technique the heritability was increased to 61%. In a separate experiment, QR heritability ranged from 43 to 71% with ascospore shower and field evaluation, respectively (Raman et al. 2018). These heritability values seemed to be ideal for GS implementation. Furthermore, GS is likely more advantageous than phenotypic selection in the long term by allowing more lines to be evaluated in a single selection period.

As LD decays more rapidly, increased marker density and training population size is required for a more accurate prediction. In this respect, prediction for individuals distantly related to the training population generally results in lower accuracy. In Fikere et al. (2018), prediction across oilseed rape subtypes (winter and spring types) resulted in a lower accuracy than when the prediction was made separately on each type. This result is consistent with that of Jan et al. (2016) who reported an increase in prediction accuracies for agronomic traits when the prediction was done on subpopulations separately. Hence, it is important to maintain a close relationship between the selection candidates and the training population, which can be done by constantly updating the prediction model whenever new phenotypic data are generated (Poland and Rutkoski 2016). As oilseed rape is characterized to have a distinguishable subpopulation structure (winter and spring type), genome-wide prediction models implemented separately for each subpopulation will likely increase the efficiency of GS for blackleg resistance.

The rapid development in sequencing is resulting in the discovery of high-quality genome-wide markers. In *Brassicas*, there are ongoing efforts to increase the quality of genomic resources by capturing as much genetic variation within the species through building pan-genomes/super pan-genomes. The availability of such genomic resources for which thousands of markers can be derived and the development of statistical models that can integrate more biological information and multi-trait data will complement GP's implementation for improving blackleg resistance and other yield-related traits in *Brassicas*.

## Conclusion

Quantitative resistance was the primary form of blackleg resistance selected in most oilseed rape cultivars before R-genes were explored for breeding. QR has been shown to afford a broad-spectrum control and its modest genetic effects render the blackleg pathogen to less likely evolve; hence, its effectiveness is more durable compared with R-gene-mediated immunity. However, the study of the underlying mechanisms through conventional genetic analysis has been hampered by its sensitivity to GxE interaction. The past decades saw an overwhelming exploration of DNA markers, through QTL mapping and GWAS to study the genetic basis of quantitative resistance against blackleg. These efforts not only resulted in the identification of several loci conferring QR in range of *Brassica* germplasm, but also uncovered the complicated genetic and diverse molecular mechanisms controlling QR. This complexity often hinders the full exploitation of blackleg QR in breeding. Recent developments in genomics along with the improvement in phenotyping approaches have helped unravel the genomic organization of blackleg resistance loci in the *Brassica* genome, resulting to the discovery of genome-wide markers that are facilitating the identification of candidate genes underlying QR through association genetics and comparative genomics. The manipulation of these genes is expected to further elucidate the underlying mechanisms of QR-mediated blackleg resistance.

Advances in genome sequencing are projected to further improve the quality of current *Brassica* genome and pan-genome references as more sequence information derived from *B. napus* lines and morphotypes is generated. These improved genome references will encompass both genic and non-genic regions in *Brassica* species, which will greatly assist in understanding the overall genomic architecture of important traits including quantitative resistance. In addition, the integration of other omics approaches, such as transcriptomics, phenomics and epigenomics will likely improve the understanding of the genetic and molecular underpinnings of quantitative resistance. When integrated with advanced statistical models, this wealth of biological information will result in a more accurate prediction of the genetic performance of lines in GS, thereby accelerating the improvement of blackleg resistance and other important agronomic traits that directly affect yield in oilseed rape.

As *L. maculans* continues to threaten global oilseed rape production due to its highly dynamic biological nature that adapts to changes in crop production practices brought about by global climate change, the need for more effective and sustainable means of disease control is becoming even more important. This can be achieved through the adoption of improved agronomic practices and systematic deployment of genetic resistance. While the deployment of major R-genes will remain an important component of blackleg management in oilseed rape, their effectiveness could be further enhanced by incorporating QR, resulting in a more durable and sustainable control strategy. Thus, the exploration and utilization of QR in oilseed rape cultivar development will become even more critical and relevant to blackleg disease management.
